# Oyster breakwater reefs promote adjacent mudflat stability and salt marsh growth in a monsoon dominated subtropical coast

**DOI:** 10.1038/s41598-019-44925-6

**Published:** 2019-06-12

**Authors:** Mohammed Shah Nawaz Chowdhury, Brenda Walles, SM Sharifuzzaman, M. Shahadat Hossain, Tom Ysebaert, Aad C. Smaal

**Affiliations:** 1 Wageningen Marine Research, Wageningen University and Research, PO Box 77, 4400 AB Yerseke, The Netherlands; 2 Aquaculture and Fisheries Group, Wageningen University and Research, PO Box 338, 6700 AH Wageningen, The Netherlands; 3 Institute of Marine Sciences, University of Chittagong, Chittagong, 4331 Bangladesh; 4 Royal Netherlands Institute of Sea Research (NIOZ) and Utrecht University, Department of Estuarine and Delta Systems, PO Box 140, 4400 AC Yerseke, The Netherlands

**Keywords:** Physical oceanography, Marine biology

## Abstract

Oyster reefs have the potential as eco-engineers to improve coastal protection. A field experiment was undertaken to assess the benefit of oyster breakwater reefs to mitigate shoreline erosion in a monsoon-dominated subtropical system. Three breakwater reefs with recruited oysters were deployed on an eroding intertidal mudflat at Kutubdia Island, the southeast Bangladesh coast. Data were collected on wave dissipation by the reef structures, changes in shoreline profile, erosion-accretion patterns, and lateral saltmarsh movement and related growth. This was done over four seasons, including the rainy monsoon period. The observed wave heights in the study area ranged 0.1–0.5 m. The reefs were able to dissipate wave energy and act as breakwaters for tidal water levels between 0.5–1.0 m. Waves were totally blocked by the vertical relief of the reefs at water levels <0.5 m. On the lee side of the reefs, there was accretion of 29 cm clayey sediments with erosion reduction of 54% as compared to control sites. The changes caused by the deployed reefs also facilitated seaward expansion of the salt marsh. This study showed that breakwater oyster reefs can reduce erosion, trap suspended sediment, and support seaward saltmarsh expansion demonstrating the potential as a nature-based solution for protecting the subtropical coastlines.

## Introduction

Coastal habitats play a critical role in coastal adaptation strategies as they can reduce the vulnerability of coastal communities to natural hazards like flooding, eroding shorelines and sea level rise^1–4^. These habitats include coral reefs^5^, reef-forming bivalves^6–9^, dense vegetation of kelps and seagrasses^10,11^, salt marsh vegetation^12–14^ and mangroves^15–18^. They have the capacity to reduce flow and dampen wave energy through their physical structures and by doing so, they trap and stabilize sediments, allowing to keep pace with sea-level rise by natural accretion and growth^13,19–23^. Moreover, they offer additional ecosystem services including: (1) water quality regulation^24,25^; (2) ecosystem succession^26,27^; and (3) fisheries production^28–30^. The use/design of sustainable ecosystems that integrate human society with related natural habitats for the benefit of both is called ecological engineering^31–33^. It provides opportunities to combine engineering principles with ecological processes to reduce environmental impacts of man-made infrastructure^34^.

The coastline of Bangladesh has changed rapidly over the last few decades^35,36^. Until 2015, a total of 1,576 km^2^ area was lost due to shoreline erosion at an annual rate of 6.3 km^2^ in 1985–1995 and 11.4 km^2^ in 2005–2015, respectively^35^. Shoreline erosion is increasingly threatening coastal communities and their livelihoods^37^, forcing thousands of people to migrate to the mainland^38^. This is particularly severe in offshore (island) areas, such as in the islands of Kutubdia and Sandwip that are frequently impacted by storm surges, increasing astronomical tides and erosive waves associated with southwest monsoon winds^35^.

Mangroves, salt marshes and oyster reefs, which form part of the biotic environment of the coastal ecosystems in Bangladesh have the ability to provide coastal protection through trapping sediments and promoting accretion. Mangroves have proven to be cost effective in dissipating wave energy and reducing hydraulic load on embankments during storm surges^39^. However, only 60 km out of a total of 957 km sea facing embankments are protected by a forest belt, which is gradually degrading due to ever increasing cyclones^40^. Moreover, mangroves were being deforested due to construction of  aquaculture ponds and salt pans in many intertidal areas^41^. The succession and growth of other vegetation types, such as salt marshes varies with season and is becoming less efficient in trapping sediments due to incoming erosive waves during high energy periods in the monsoon season (see Supplementary Fig. S1).

Oyster reefs form persistent, three-dimensional structures which can attenuate waves^27,42^, trap sediment^7,9,43,44^, and are resilient growing with sea level rise^22,23^. Moreover, it provides additional ecosystem services, such as habitat for fish and resident invertebrates^28–30,45,46^, improve water quality and enhance primary production^24,47,48^. Oyster reefs also provide opportunities for oyster aquaculture by increasing seed supply to oyster culture areas^49^. However, the effectiveness of oyster reefs in coastal protection has not yet been tested in the context of monsoon-dominated subtropical coasts, such as Bangladesh.

This experimental study investigated the scale of morphological changes after constructing replicated (three) oyster breakwater reefs on an eroding intertidal mudflat of the Kutubdia Island at southeast Bangladesh. We evaluated the hypothesis that wave attenuation by these oyster breakwaters could reduce sediment erosion, promote mudflat stability, and enhance lateral saltmarsh expansion and growth. Oysters occur abundantly in the study area and the intertidal rock oyster, *Saccostrea cucullata* is the dominant species found on all types of hard substrates, i.e. on oyster shells, boulders, sluice gates and jetties pilings.

## Results

### Wave dissipation

**Figure 1:**
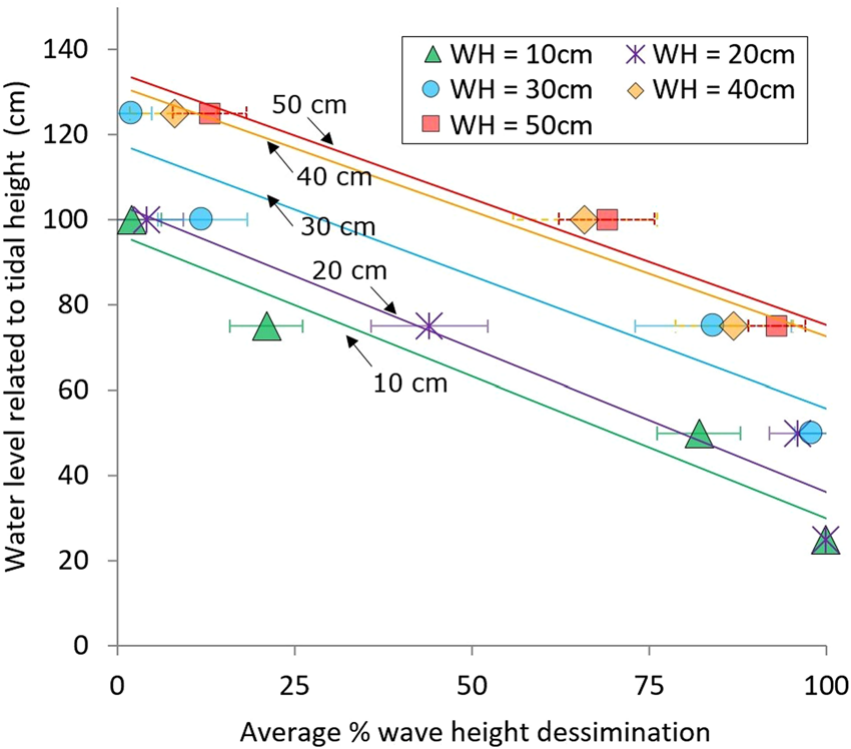
Percentage of wave height dissemination by the reefs, as measured at five water levels related to tidal height (25, 50, 75, 100 and 125 cm) of wave measurement points, classified in five different wave heights (WH). Lines show the linear regressions for each wave height. They indicate the reduction of wave height in relation to increase in tidal (water) levels.

The reefs, being ~0.6 m in height after settling above the mud, dissipated wave energy, acting as wave breakwaters. Dissipation of wave energy depended on tidal height (water level) and wave height (Fig. 1). Wave heights varied per season, and small waves (<20 cm) were recorded in post-monsoon (Oct-Nov) and winter (Dec-Feb) seasons. The highest wave height (~50 cm) was observed in pre-monsoon period (Mar-May), when most of the tropical depressions appeared in the Bay of Bengal. Wave heights ranged from 20–40 cm during monsoon period depending on wind speeds. Waves were blocked (i.e. attenuated) 95–100% by the vertical relief of the reef at water levels <0.5–0.6 m. At water levels between 0.6–1.0 m, waves broke and dissipated depending on water level and wave height (Fig. 1). For water levels >1.0 m, the smaller waves were not dissipated by the reef structures, whereas the larger waves (40–50 cm) were still dissipated.

### Seasonal sediment dynamics

**Figure 2:**
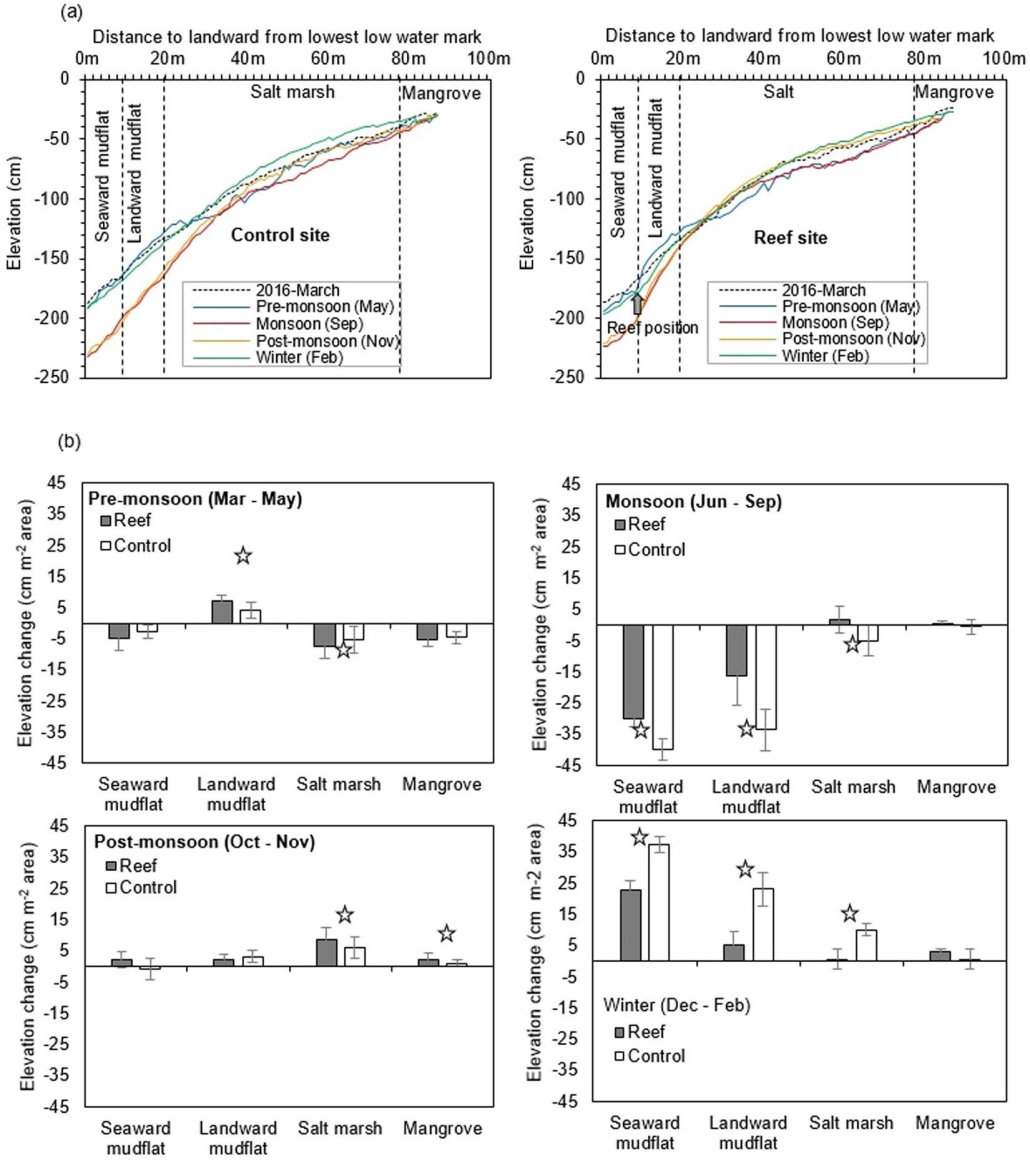
(**a**) Elevation profiles, based on seasonal measurements from March 2016 to February 2017, along three transects crossing the constructed reefs (right) and two transects crossing control sites (left) without any reefs, during the pre-monsoon, monsoon, post-monsoon and winter; (**b**) comparison in changing elevation at seaward mudflat (0–10 m), landward mudflat (10–25 m), salt marsh (25–80 m) and mangrove (>80 m) areas of reef and control sites by the end of pre-monsoon (May 2016), monsoon (September 2016), post-monsoon (November 2016) and winter (February 2017). Elevation changes were the difference between two consecutive seasons, whereas changes in post-monsoon were measured by the differences between the initial transect profile surveyed in March 2016 and the transect profile of May, 2016. Star on the bar showing significant difference in reef and control sites.

The overall site showed complex sediment dynamics as influenced by four different seasons (Fig. 2a,b). Seasonal transect measurements at the control and reef sites indicated that during the pre-monsoon period (March-May) erosion occurs at both the vegetated (i.e. mangrove and salt marsh) areas and seaward mudflat areas. Whereas significantly (*p* < 0.001) higher sedimentation occured at the landward mudflat of reef sites than the control sites (Fig. 2b). Possibly sediment eroded from the supralittoral zone was captured in the upper mudflat area of the reef sites. Southwest monsoon winds became active from May causing maximum rainfall (2,162 mm) during the monsoon period (June-September) in 2016. The rain showers, coupled with higher waves generated by southwest monsoon winds triggered greater erosion along the transect at the control sites (Fig. 2a). At the reef sites, erosion was observed also in the seaward and landward mudflat area during monsoon (Fig. 2a), but was significantly (*p* < 0.001) less as compared to control mudflats areas (Fig. 2b). For both control and reef sites, the highest erosion rates occurred in the lower portion (seaward) of mudflat during the monsoon, though the erosion was still significantly (*p* < 0.001) lower at reef sites. Sediment deposition occurred during the post-monsoon (i.e. November) predominantly in the vegetated areas at both control and reef sites (Fig. 2b). Sediment deposition was significantly (*p* < 0.001) higher at the saltmarsh areas of reef sites than at the salt marsh areas of the control sites during this period. In the mudflat area (i.e. seaward and landward mudflat) no distinct changes were observed in the post-monsoon season and elevation remained identical at the end of the monsoon period (Fig. 2a). By the end of the winter (i.e. February 2017) sediment accumulation reached maximum levels along the transects both at the reef and control sites with elevation level as higher as compared to March 2016. Sedimentation was maximum in the lower (seaward) portion of the mudflat for both the control and reef sites. Despite the presence of the reef structures, elevation along the transects for both control and reef sites appeared to be similar in February 2017. These observation indicate that tidal flat morphology is in a dynamic equilibrium, with high erosion during the monsoon period, followed by deposition during the dry winter.

### Changes in tidal flat morphology

**Figure 3:**
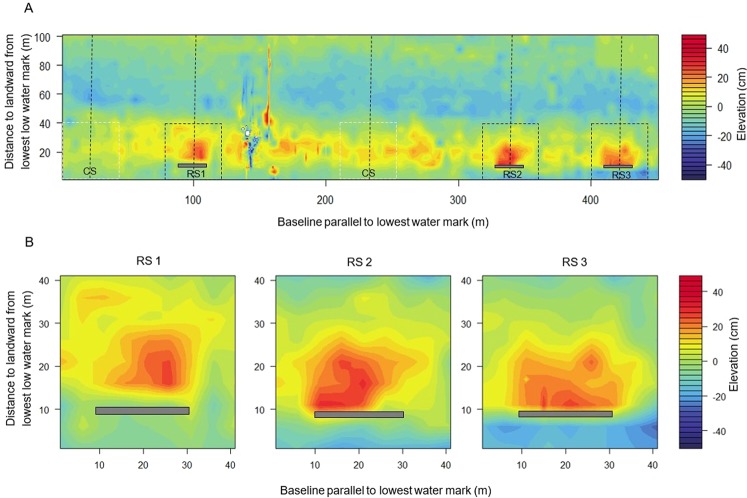
Changes observed for mudflat elevation areas around the oyster breakwater reefs. (**A**) Showing the differences in elevation between October 2015 and October 2016; the position of reefs (RS, grey bars) and control sites (CS). (**B**) Zoom (40 m × 40 m) in view on RS1, RS2 and RS3 to observe the sediment accreted areas around the breakwater oyster reefs (grey bars).

**Figure 4:**
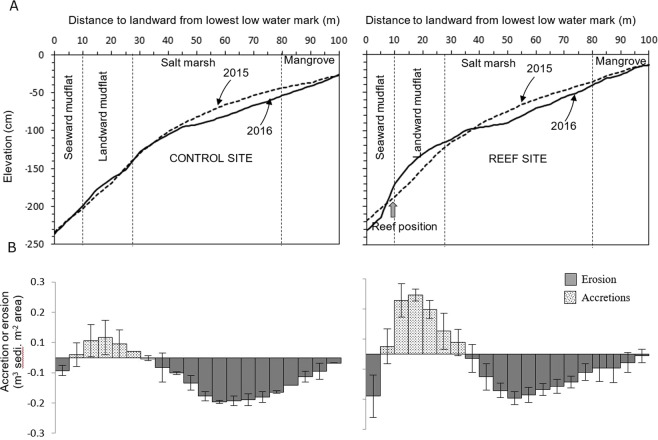
(**A**) Mean elevation change across the transect lines over one year period, and (**B**) net sediment accretion or erosion rates by distance classes along the reef (right) and control (left) sites.

Based on the seasonal sediment dynamics observed above, the difference map between October 2015 (before reef deployment) and October 2016 (after reef deployment, Fig. 3) describes the effect of reefs on the erosion and deposition patterns in monsoon season. The difference map (Fig. 3) shows that reefs locally had an impact on the tidal flat morphology. For example, in the low intertidal areas significant accretion (*p* = 0.03) occurred up to 35 m landward of the reefs with a maximum accretion of 29 ± 1 cm (Fig. 4). Accretion was also observed at the low intertidal areas of control sites, but was only 12.5 ± 4.5 cm. Beyond 35 m landward of the reefs, sediment heights were similar at reef and control sites (Fig. 4).

Average sediment accretion or losses along cross sections of the difference map indicated an average accumulation of 0.11 m^3^ sediment/m^2^ area that occurred over an area ranging 5–35 m at the landward of the reef sites (Fig. 4), and this rate was 3x greater than those measured for the control sites (0.03 m^3^ sediment/m^2^ area). On the other hand, erosion occurred at distance class between 0–5 m and 35–100 m along the assessed cross-sections for both reefs and control sites (*p* = 0.32) over a 12 months period. Cumulative changes in terms of either the erosion or accretion rates along the whole transect (0–100 m) demonstrated that erosion on average are dominant at both reef and control sites, but at different rates. Average net erosion rates are almost 2x higher over the entire transect at control sites (0.051 m^3^ sediment m^−2^) vs. at the reef sites (0.023 m^3^ sediment m^−2^).

### Sediment grain size

Sediment at the study site mainly consists of clay particles (>84%) (see Supplementary Fig. S3). The % clay changed seasonally under the influence of the monsoon winds and waves. During the low energy periods (November-February), the sediment consisted of ~96% clays with the accumulation of finer sediments, whereas sediment contained less (~84%) clay during the monsoon months (June-October) when intensity of water turbulence, and surficial erosion rates were high due to heavy rainfall and flash-flooding events. Before reef deployment, all of sites showed similar characteristics in grain sizes (see Supplementary Fig. S4). After reef deployment, fine sediments were trapped by the breakwater reefs due to changes in local hydrodynamics resulting in a higher percentage (95 ± 3%) of clay at leeward of the reefs as compared to the control sites (90 ± 6%). This difference was more prominent (92% reef vs. 84% control) during the monsoon months, but it was not significantly different (*p* > 0.05). The difference in clay percentage seaward of the reefs vs. the control sites was minimal (*p* = 0.32).

### Lateral salt marsh movement

**Figure 5:**
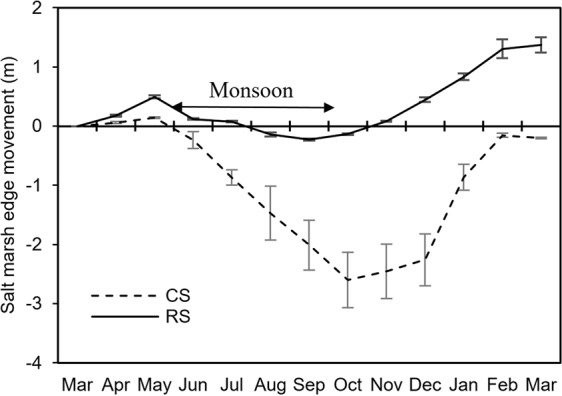
Seasonal dynamics in the movement of salt marsh edge at control (CS) and reef (RS) sites.

**Figure 6:**
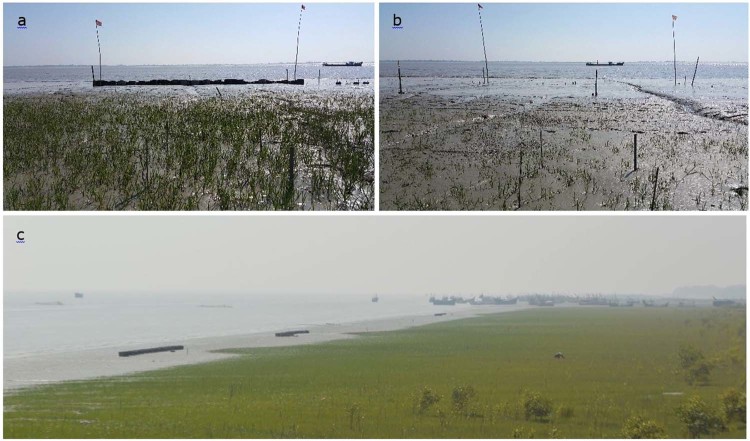
Photographs showing the difference in salt marsh growth at (**a**) reef and (**b**) control sites in December 2017, and (**c**) seaward salt marsh expansion (photograph was taken in February, 2019).

Lateral salt marsh movement (i.e. seaward expansion or landward retreat) showed a seasonal pattern (Fig. 5). During the monsoon (when erosion process dominated on the mudflat) the salt marsh retreated, whereas at the end of the winter (when sedimentation processes dominated on the tidal flat) seaward salt marsh expansion was observed. Retreatment rate of the saltmarsh during the monsoon was significantly higher (*p* < 0.05) at control sites as compared to the reef sites (Fig. 6). During the dry winter months a faster seaward expansion of the marsh was observed at the reef sites as compared to the control sites. Overall, a seaward salt marsh expansion of 1.37 ± 0.13 m was observed at the reef sites in one year post-construction of the oyster breakwater reefs, whereas the salt marsh seaward margin retreated 0.20 ± 0.01 m at the control sites.

Salt marsh stem density also declined during the monsoon, with highest rates of loss at the control sites (CS 54% vs. RS 42%). Saltmarsh started to expand again in the post-monsoon season, while the steam density increased, reaching maximum during the winter months (see Supplementary Fig. S5). Salt marsh regeneration rates (increase in stem density m^−2^ area) were about 36% higher at reef sites than that of the control sites (see Supplementary Fig. S5).

## Discussion

The tidal flat investigated in this study showed complex seasonal sediment dynamics. Sediment deposition was common during winter (December - February) period due to low energy incoming waves^50^. A dry (<60% humidity) and hot (~4 °C above the average) air from the north western India moves into the northern Bay of Bengal during the pre-monsoon (March-May) season causing a pre-monsoonal depression in the region^51^. At that time the coast receives strong longshore currents and waves. As a result, deposited sediments begin to erode from intertidal flats. Sediment erosion reaches a maximum rate during the monsoon (June-September) season, when southwest monsoonal winds result in huge rains and much more turbulent waves^52^. Subsequently, new sediments deposited in the late post-monsoon season (November), that continues throughout the dry winter season. The tidal flat seems unchanged at the end of the winter as compared to the previous winter period (Figs 4A, 5, see transect line March 2016 and February 2017), indicating that the sediments lost in the previous seasons were regained. As more sediment eroded at the control sites, sedimentation rates were maximum during the winter as more sediment was needed to compensate for the higher losses caused by the monsoon. With only a single year of data, it is difficult to say whether the tidal flat investigated is in a dynamic sediment equilibrium.

If we focus on the effect of the monsoon season alone, sites reach their highest erosion state, oyster breakwater reefs appear to be effective locally at reducing tidal flat erosion (Figs 3 and 4). The deployed structures enhanced sediment deposition at the leeward (landward) side of the reefs, increasing the bed level up to 29 cm. Interestingly, the control sites also showed some deposition (<12 cm) in landward mudflat portion (see Fig. 4) and the mechanism is unknown, but it could be a common topographic cycle at the site. Still the sedimentation rate is significantly higher in all reef sites than controls, confirming the morphological effect of the breakwater reefs. Furthermore, sediment composition changed post-deployment of the breakwater reefs. Notably, mean sediment grain sizes were more stable, even in monsoon months, with finer sediment (i.e., clay and less sand) as compared to the control sites (see Supplementary Fig. S4). Changes in deposition and sediment grain characteristics are a result of probably the interaction between hydrodynamics (i.e., waves action), and the relative height of the breakwater reefs. Wave dissipation was noted when the water level is less than a meter above the reefs. If the wave height was smaller than the relative water depth above the breakwater reefs, wave dissipation was minimal. Despite the importance of reef structures to attenuate wave energy, almost no data on wave dissipation can be found in literature. Our study shows for the same relative water level that larger waves are dissipated more by the reef than smaller waves. La Peyre *et al*.^53^ observed that reefs were more effective in reducing shoreline erosion in an exposed area compared to a sheltered area. The interaction of a reef with larger waves could explain for this difference. Waves and wave dissipation are the primary reasons for the observed local morphological changes. Our study clearly indicates that breakwater reefs can successfully reduce the wave heights (i.e. wave energy). It provided better stability in sediment movement from intertidal bed and reduces erosion, which was not the case for control site without reefs. A 50 cm vertical relief of breakwater reefs resulted in long-periods of reduced wave height, resulting in an area of influence over 30 m behind the reef favoring sediment accumulation. Moreover, this accumulated sediment, which normally erodes during the monsoon months, was successfully trapped behind (lees) the reefs, thus reducing erosion and stabilizing the tidal flat locally. However, these effects might vary, depending on the intensity of monsoon. The net sediment budget at the end of monsoon (i.e. October) after 2016, but before 2015 also indicated that the oyster breakwater reef could reduce erosion rate by ~50% (i.e. 0.014 m^3^ sediment/m^2^ area) along the entire reef cross-sectional tidal flat area than the adjacent control areas (see Fig. 4). Though winter deposits resets the whole area, with no reef effect visible during winter, this situation only lasts for three months (December-February), while the rest of the year tidal flat showed positive effect on sediment state. Morphological changes in the intertidal mudflat due to reef placement might also depend on other factors. For example, in the Oosterschelde (SW Netherlands), Walles *et al*.^9^ found that the leeward side of constructed oyster reefs was elevated due to sediment accumulation and the effect was correlated with the reef dimension (length). They suggested that longer extended reefs were more likely to generate larger impacts on tidal flat morphology. The experimental reef units used in this study were only 20 m in overall length resulting in an impact on the mudflat morphology up to 30 m area at the landward side. Beyond this distance, erosion was unchanged for reef and control sites. This entire zone was severely affected continuously by wave actions particularly during the monsoon period. The effect of the reef could be enhanced by extending the length and height of the reefs depending on immersion time in the investigated site. Moreover, the extent of the impact also depends on tidal range, wave condition, bed slope and flow directions at the reef construction site^54^. Studies^43,55^ along US coast have indicated that the effect of reefs in controlling shoreline erosion is quite variable over time, with their efficacy only viewed as significant during storm induced erosion. However, this study showed that oyster breakwater reefs were equally effective in trapping sediment for all seasons including stormy periods (for instance, a tropical cyclone with wind speeds of 70–110 km h^-1^, named ‘Roanu’ that hit the study site on May 21, 2016).

Not only the waves, regular flow and current dynamics around oyster reefs have also been shown to be crucial in driving local sedimentation^56,57^. As particulate-laden water moves over an oyster reef, eddies slow water flow to the point where deposition may occur, and the amount of deposition dependent upon suspended sediment concentration in ambient water and their particle size and flow speed. Suspended sediment concentration were higher in ebb than the flood tidal phase, but the tidal current was relatively stronger in ebb tide compared to flood tide, suggesting that sediment deposition rate varies with the tidal phase. Longshore flow and current velocity showed some variation in and around the reef areas. Depth-averaged mean current velocity was less strong in the landward side of the reef than the seaward side^58^. Whitman and Reidenbach^59^ conducted a study of flows along mudflats and found that flow velocity was reduced by a factor of 2 compared with flows over an adjacent oyster reefs. In this study, high sediment deposition behind the reefs suggest these low flow areas tended to trap fine sediments. However, drag exerted on current flow by an object like oyster reefs and its influence on sediment deposition and particle flux are a function of the length and orientation of that object in the direction of the flow^60^. Perpendicular reefs were found more effective than parallel or circular reefs at producing hydrodynamic conditions that maintain higher deposition rate^56^. In this study, the reefs were placed parallel to coast as breakwater for attenuating wave energy that also effective in trapping sediment. We did not see any possibility that the placement of the parallel breakwater reefs accelerates erosion in adjacent areas including the control sites.

In monsoon season, water flows were strong and erosion of other intertidal ecosystem like salt marshes occur. Short-distance interactions between the reef structures and adjacent salt marshes could accelerate the process toward more local tidal flat stability^61^. As indicated above, reefs can influence the tidal flat morphology during the monsoon period, impacting nearby salt marsh vegetation in terms of increased plant survival and lateral marsh expansion (Figs 5 and 6). At both the control and reef sites, seasonal dynamics in terms of the lateral salt marsh movement showed retreat during the monsoon followed by seaward expansion. Overall landward salt marsh retreat was significantly lower at the reef sites as compared to the control site during the monsoon season. By reducing the overall erosion rates at landward (= behind the reefs) area, the reefs have created a window of opportunity for salt marshes to withstand pressure of erosion during the monsoon season, facilitating its seawards expansion, 1.37 ± 0.13 m yr^−1^ vs. −0.20 ± 0.01 m yr^−1^ (i.e. retreat) in control site. Alteration of the physical conditions by the deployed reef structures protected the salt marsh during the monsoon season, resulting in the seaward expansion and fast regeneration of the marsh after the monsoon season. Previous studies have only recorded a reduction of marsh edge erosion after deployment of the oyster reefs^43,53,62^. This is the first study from subtropical region showing seaward expansion of salt marshes after deployment of reef structures. Furthermore, a much larger area can be influenced and protected using longer reef structures and increasing the vertical relief that, in turn, can elevate the intertidal bed and create other important shallow habitats, like mangroves^63,64^. Breakwater structures have often found to play a successful role to rehabilitating mangrove forests in subtropical regions^65^. Therefore, oyster breakwater reefs not only have the potential for stabilizing the tidal flats but also useful for restoring or enhancing other ecologically important habitats such as salt marsh, offering favorable conditions for many organisms to dwell and grow (so-called Window of Opportunity)^66^.

Apart from the investigated Kutubdia Island, breakwater reefs can also be implemented in other coastal areas of Bangladesh where natural habitat of oysters is present. Oyster settlement and growth, in general, are characterized by relatively high levels of salinity, Chlorophyll-a, dissolved oxygen and pH. Therefore, estuaries dominated by freshwater plume and coastal areas with high suspended sediments are less suitable for oysters^58^. For example, the central and southwestern coasts of Bangladesh may not be ideal for deploying artificial oyster reefs as these areas are prone to large amount of sediment-laden freshwater discharge through the Ganges-Brahmaputra-Meghna (GBM) river system. While ~397 km coastline and offshore islands of the southeastern coast, which receives much influence from the Bay of Bengal than other parts and blessed with plentiful oyster spat supply, are deemed suitable for year round growth of oysters^58^. The development of oyster reefs is thus feasible in those areas to improve salt marsh and mangroves ecosystems for generating multiple ecosystem services and reducing the vulnerability of coastal islands from erosion.

In conclusion, the use of oyster breakwater reefs at lower intertidal zone can protect the tidal flat in front of primary embankments by changing the shoreline eco-morphology. The results of this study demonstrated that oyster breakwater reefs are particularly useful in reducing erosion at lower intertidal areas as the reefs successfully stabilized sediments in both high (monsoon) and low energy periods. Additionally, they enhanced the growth of adjacent salt marsh vegetation, which expanded their seaward edge thereby further stabilizing the adjacent unconsolidated sediments. This effect can be improved by widening the reef length and height. Moreover, the reef structures provide space for new oysters to grow and develop as biogenic habitat overtime leading to a self-sustained oyster reefs. Therefore, along the coast of Bangladesh, where larval supply of oysters are abundant, the eco-engineered breakwater structures hold promise for a more sustainable shoreline protection against erosion.

## Methods

### Study area

**Figure 7:**
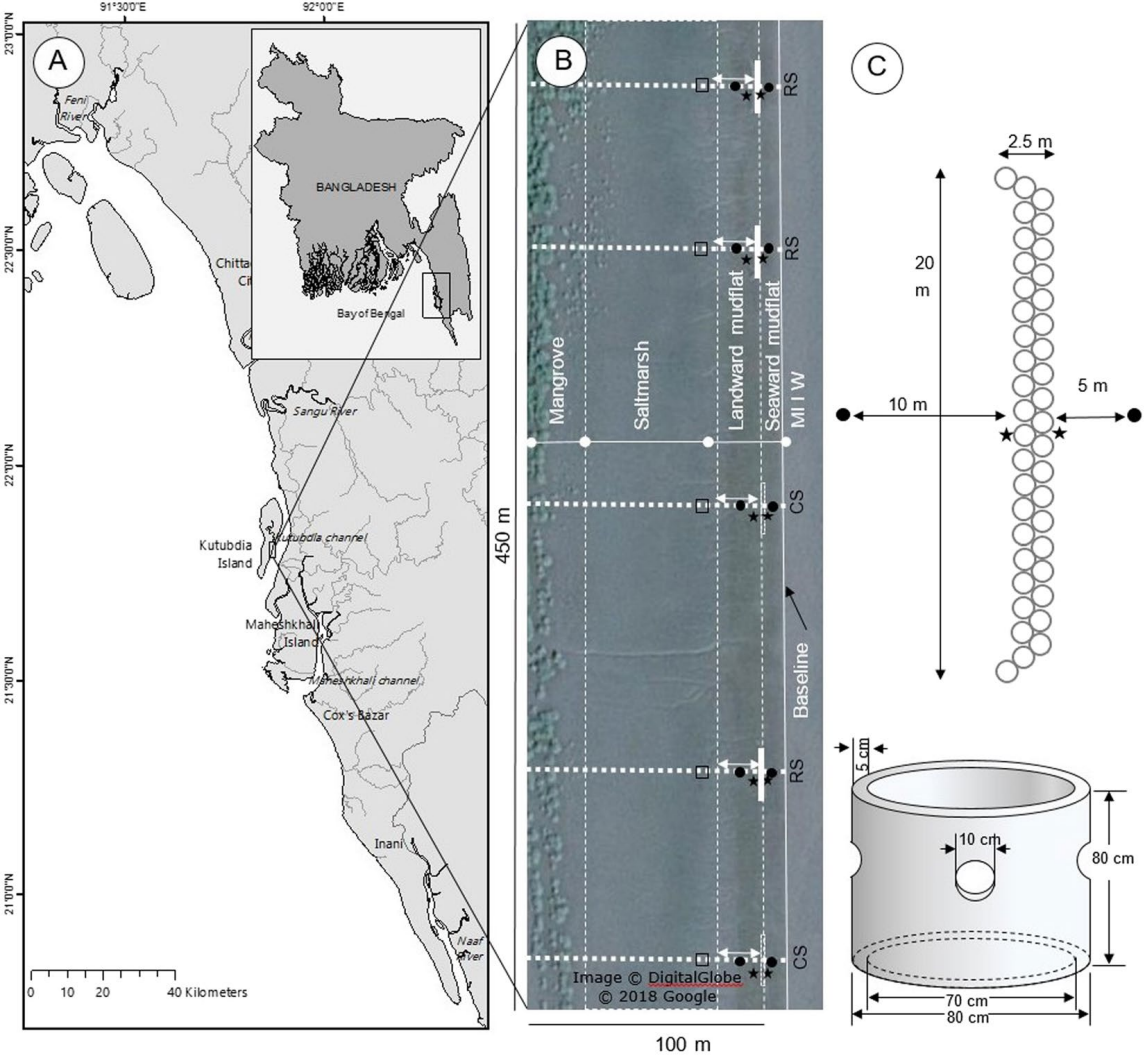
(**a**) Study area in the Kutubdia Island, Bangladesh; (**b**) Google earth satellite image (2017) showing the experimental reef sites (RS = reef site; solid white rectangles), and control sites (CS = control site; dotted white rectangle) with ecological settings (mudflat, salt marsh, mangroves) of the area. Thick dotted white lines were transect lines for measuring monthly and seasonal changes of shore profiles; Black dots were sediment sampling stations; Black stars were wave gauges; White arrows were marsh retreat/seaward expansion monitoring points, measuring the distance between a benchmark stick at the reef edge and the salt marsh edge. Black square: quadrates for salt marsh density measurement; (**b**) Reef dimension. A 20 m long reefs was constructed by placing 41 concrete rings in two rows next to each other.

A manipulative field experiment was carried out at Kutubdia island (Fig. 7), located in the southeast coast of Bangladesh. Over the last 42 years (1972–2014) erosion rates have increased reaching up to 33.7 m yr^−1^ ^67^. About 40 km of earthen embankments, including 4 km of concrete blocks, have been constructed since the 1990s to protect the island, although a large area still unprotected and exposed to tidal flooding and erosion. The earthen embankments often collapse and cannot prevent flooding during the monsoon period, and require maintenance every year with new alignments as there is a constant loss of the foreshore. The east part of the island, which is characterized by wide tidal mudflats, is a suitable habitat for saltmarsh and mangroves^68^. Currently, 290 ha area are vegetated by mangroves/salt marsh covers, which is only 4.2% of the total island area^69^. Oysters occur along the shoreline where substrates are available.

A field experiment was carried out at the Boroghop jetty site at the eastern side of the island (Fig. 7). Here the mudflat slope is gentle (~1.4°). The southwest and northeast monsoon winds together with north-easterly winds in non-monsoon periods govern the prevailing four different seasonal weather patterns, i.e., winter (December-February), pre-monsoon (March-May), monsoon (June-September), and post-monsoon (October-November) periods in the coastal areas of Bangladesh^70,71^. In the study area, the climatic conditions are mild and dry during winter, with air temperature ranging from 6.2–22.4 °C. Winds are north-easterly at the beginning of winter but become north-westerly by the end. Air temperature is maximum (~38.5 °C) during the pre-monsoon season. Heavy southwest monsoon rains begin in early June, continuing in to mid-October. During the monsoon season, floodwaters from rainfall lowers the salinity to estuarine conditions (10–15 ppt). Salinity levels in other seasons, including the post-monsoon season remain steady, >22 ppt. Annual average rainfall varies from 2,300–3,200 mm^52^. Suspended sediment concentrations in the water are quite high, varying among seasons from 100–700 mg l^−1^ ^72^. Tides along the coast are semi-diurnal, and the tidal range is approximately 4 m with a seasonal variation of mean tide level (MTL) 50–80 cm. Water current direction is from the north during peak flood tides and from the south during ebb tides. During the pre-monsoon and monsoon seasons strong longshore currents prevail^50^ with high and variable waves because of summer storms, reaching a height of 0.75 m or more depending on wind conditions. Average annual wind speed ranges from 0.8–2.2 m sec^−1^.

### Constructed oyster breakwater reefs

**Figure 8:**
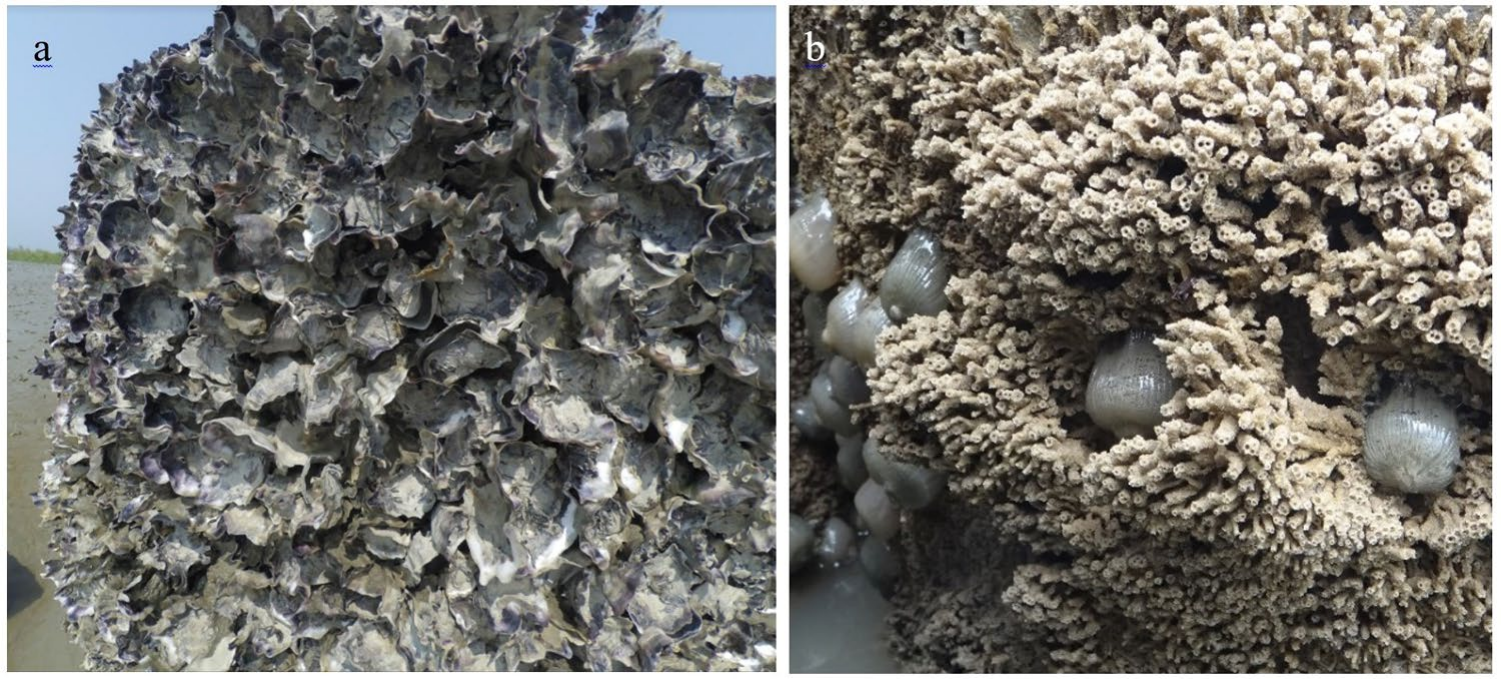
Abundance of: (**A**) oysters; and (**B**) reef forming polychaetes (*Sabellaria* sp.) with anemones in the upper (top 50 cm) and lower (bottom 30 cm) parts of the oyster breakwater reefs, respectively. Photographs were taken in October 2017, twenty months after deployment of the concrete rings.

Oyster reefs were often constructed by using shell derived materials, forming either piles of loose shell or in bags or filling gabions with loose shell^27,73,74^. In a dynamic and high energy coast, like the study site, more robust reefs are needed with high vertical relief to avoid smothering by sediments and less physical damage to the deployed structure during the monsoon season^6^. Three oyster reefs were constructed on a tidal mudflat of Kutubdia Island using precast concrete rings. Each of concrete ring was 0.8 m in diameter, 0.8 m high, and 0.05 m thick with four holes in them^50^, a structure similar to reef balls^75^. Each reef contained 41 concrete rings, each placed in two rows next to each other, resulting in 20 m long reefs (Fig. 7). These reefs were deployed parallel to the coastline (~0.5 m above mean lower low water, MLLW) as wave-break structures to attenuate wave energy. About 50–70 cm of the rings were exposed to the air or water depending on the season and tidal phase, while rest of part (i.e. bottom side) were sunk in mud after deployment at the experimental site. Prior to the deployment of the reefs, ECOBAS project used the concrete rings on the intertidal mudflat adjacent to the experimental site (at the same tidal exposure) for two years to allow oyster larvae settlement and grow^50^. During the first year, settlement of oysters was low (<100 spat m^−2^). However, successful spat fall (>300 spat m^−2^) was observed in year two, when rings covered with high densities of oysters *S. cucullata* (~1200 individuals m^−2^; size class 5–47 mm shell length) and other marine organisms such as barnacles, sea anemones, gastropods and polychaetes. The overgrown rings were transported to the experimental site in March 2016 and termed as “oyster breakwater reefs”. A terminology OysterBreak^tm^ was also used for a similar experimental setup in Vermilion Cove, Louisiana, USA^27^ (La Peyre *et al*., 2017). Top 50 cm of the reef substrates were covered with as thick as ~10 cm layer of live and dead oysters, while the dynamic bottom part (30 cm) was occupied by various benthic epifauna, mostly reef forming polychaetes, *Sabellaria* sp. (Fig. 8).

### Wave dissipation

To quantify wave dissipation by the reef structures, wave heights were measured 0.5 m seaward and 0.5 m landward of a given constructed reef (Fig. 7). Wave dissipation was determined as the difference in wave height seawards and landwards of the reef. Wave heights were measured using a manual wave gauge which was composed of four connected sticks, equipped with a vertical ruler (cm), with a float (ping-pong ball) in between that could freely floats atop of the surface water. Vertical movements of the float (rising with the wave crests and falling with the troughs) were visually recorded monthly for 5–10 minutes at 25, 50, 75, 100 and 125 cm tidal heights for each of the three replicated reef sites. For logistic reasons, wave dissipation could only be determined for one reef site per tide. Prior to each final measurement, uncertainty in measuring wave heights were checked by setting multiple wave gauges (n = 3) parallel to each other at same depth and collecting wave height data for same waves. Any significant variation was not noted for each measurement (see Supplementary Fig. S2). Due to the large (seasonal) variation in the wave climate and measurement uncertainty, monthly data of all three reefs were first categorized according to wave heights and then only the wave heights of 10, 20, 30, 40 and 50 cm were evaluated for calculating the mean wave dissipation percentage. Ten observations for 10, 20, 30, 40 and 50 cm high waves were considered at five different tidal heights (i.e. 25, 50, 75, 100 and 125 cm), thus a total of 250 observations were evaluated from seaward and landward wave gauges to determine the mean wave dissipation rate.

### Changes in tidal flat morphology

Shore elevation was measured (March 2016 to February 2017) along a transect crossing each of the three reefs (here after called reef sites, RS) and along two transects crossing two adjacent control sites (CS) (Fig. 7). Each transect started at a location 10 m seaward of the reefs on the mudflat and proceeded to the edge of the mangrove forest 90–100 m landward of the reefs, crossing the seaward mudflat, landward mudflat, saltmarsh and mangroves. Elevation measurements were taken at <1 m intervals to capture the major morphologic changes along the transects. Elevation measurements were made with a shore-based rotating laser (TOPCON RL-H4C, 600 rpm), tripod stand and a level sensor (LS-80L) with a scale (TOPCON Corporation, Japan). The laser had a range of 1000 m and an accuracy of ~1 mm per 20 m. Since the relative distance between the mudflat and the horizontal plane of the laser was measured, a correction using a reference point with a known elevation was needed. For this purpose, 24 benchmarks referenced to national datum (i.e. mean lower low water, MLLW) were placed in the field prior to the measurements. These benchmarks consisted of a pvc pipe, filled with concrete and fixed in 0.5 meter of concrete base to a depth of 1 m into the mud still it reached the sandy under layer ensuring they would remain in place. All benchmarks were marked at the same height in the horizontal plane. Tests over time showed that the benchmarks remained at the same vertical elevation. To observe the seasonal sediment dynamics, elevation surveys were conducted at the end of each season (May: pre-monsoon; September: monsoon; November: post-monsoon; and February: winter). Survey began in March 2016 with relative height changes assessed monthly and averaged for the three reef transects. The same being done for the two control sites.

Additionally, elevation was measured on a 1 m by 1 m grid, covering the entire study area (450 m × 100 m = 45,000 m^2^), before (2015) and after (2016) the construction of the reefs. These measurements were conducted in October at the end of the monsoon season after which the maximum erosion was observed. A digital elevation model (DEM) was made in R software (version 3.3.0) using the packages grDevices and graphics. Based on the measured relative elevations, net sediment accretion or erosion could be calculated by subtracting the DEM for 2016 from 2015. To calculate net sediment accretion or erosion in terms of sediment volume (m^3^) for either the reef and control sites, five-cross sections, three crossing the middle of the reef area and two crossing the control area from baseline till the edge of the mangrove forest, were made from the DEMs of 2015 and 2016. In this regard, a 1 m wide grid pattern was used to calculate sediment accretion/erosion in terms of volume (m^3^) by multiplying the sediment height increase/decrease (in meters) and the area of corresponding grid (1 m^2^) in each cross section. Cross sections for RS and CS were averaged for each year, with changes in sediment accretion/erosion (m^3^ sediment m^−2^ area) along the cross-sections measured by comparing the year 2016 with 2015.

### Sediment grain size

To investigate sediment granulometry changes, monthly sediment samples were taken 5 m seaward and 10 m landward of each reef and at the same height at the control sites (Fig. 7). Measurements were done before (September 2015 to February 2016), and after reef construction (Mar 2016 to Mar 2017) using a core sampler (10 cm diameter). A total of 190 samples (2 samples ×5 sites ×19 months) were taken from the top 10 cm of sediment. Collected sediment samples were air dried and grounded to fine particles using a mortar and pestle. The materials were oven dried at 110 °C until constant weight. The distribution of particle sizes >75 µm were determined mechanically (sieving following the ASTM standard test method D422-6^76^), while particle sizes <75 µm retained on the sieve (No. 200 mesh) were determined by a sedimentation process, using a soil hydrometer (ASTM 151 H, USA). The soil was classified by plotting the particle-size fraction (sand: 0.05–2.0 mm, silt: 0.002–0.05 mm, and clay: <0.002 mm) in the USDA textural triangle system^77^.

### Lateral saltmarsh movement

Reference points were set at the lee side of the reefs and at the same height at the control sites by placing benchmark sticks in the middle of the reef to measure lateral (i.e. seaward expansion or landward retreat) salt marsh movement (Fig. 7). To determine the salt marsh edge movement rate, the distance between the benchmark sticks and the actual saltmarsh edge was measured every month after the reefs were set in place (i.e. March 2016) and then compared with the two control sites. Salt marsh stem density (number of stems m^−2^) was counted monthly in a fixed quadrat (1 m^2^) 15 m from the leeside of the reefs, which was initially 2–3 m inside the salt marsh edge.

### Statistical analysis

The statistical difference in net sediment accumulation or loss (m^3^), mean clay percentages, salt marsh stem density at reefs and at the control sites were verified, using a simple t-Test. Before statistical analysis, the normality of response variables was tested using the Kolmogorov-Smirnov Test, and a homogeneity of variances using Levene’s Test. All analyses were performed using IBM SPSS statistics software (Version 2015) by setting statistical significance at *p* ≤ 0.05.

## Supplementary information

Supplimentary information
