# Supplementary material for: Oyster breakwater reefs promote adjacent mudflat stability and salt marsh growth in a monsoon dominated subtropical coast
Source: Sci Rep. 2019 Jun 12;9:8549. doi: 10.1038/s41598-019-44925-6 (PMC6561949; doi:10.1038/s41598-019-44925-6)
Supplement: Supplementary file 1 — Supplimentary information [file 41598_2019_44925_MOESM1_ESM.docx]

**Breakwater reefs promote adjacent mudflat stability and saltmarsh growth in a monsoon dominated subtropical coast**

Mohammed Shah Nawaz Chowdhury^1,2,3,*^, Brenda Walles^1^, S.M. Sharifuzzaman^3^, M. Shahadat Hossain^3^, Tom Ysebaert^1,4^, Aad C. Smaal^1,4^

**Supplementary Information**


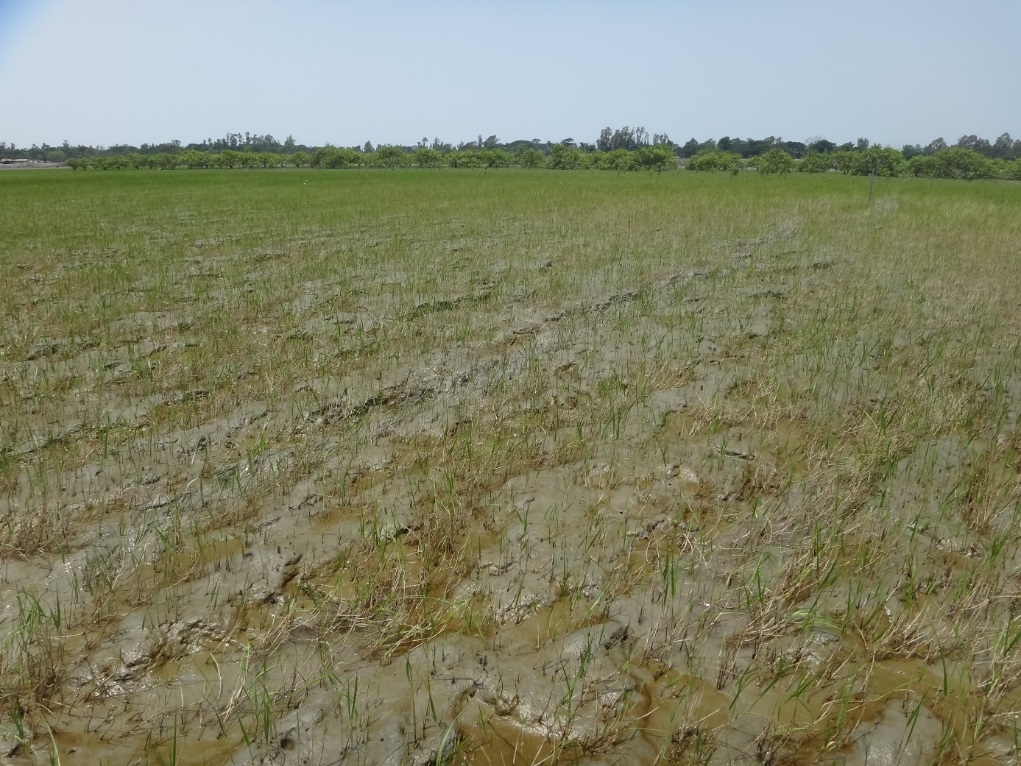


**Figure S.1** Erosion in a salt marsh during the early monsoon period at Kutubdia.


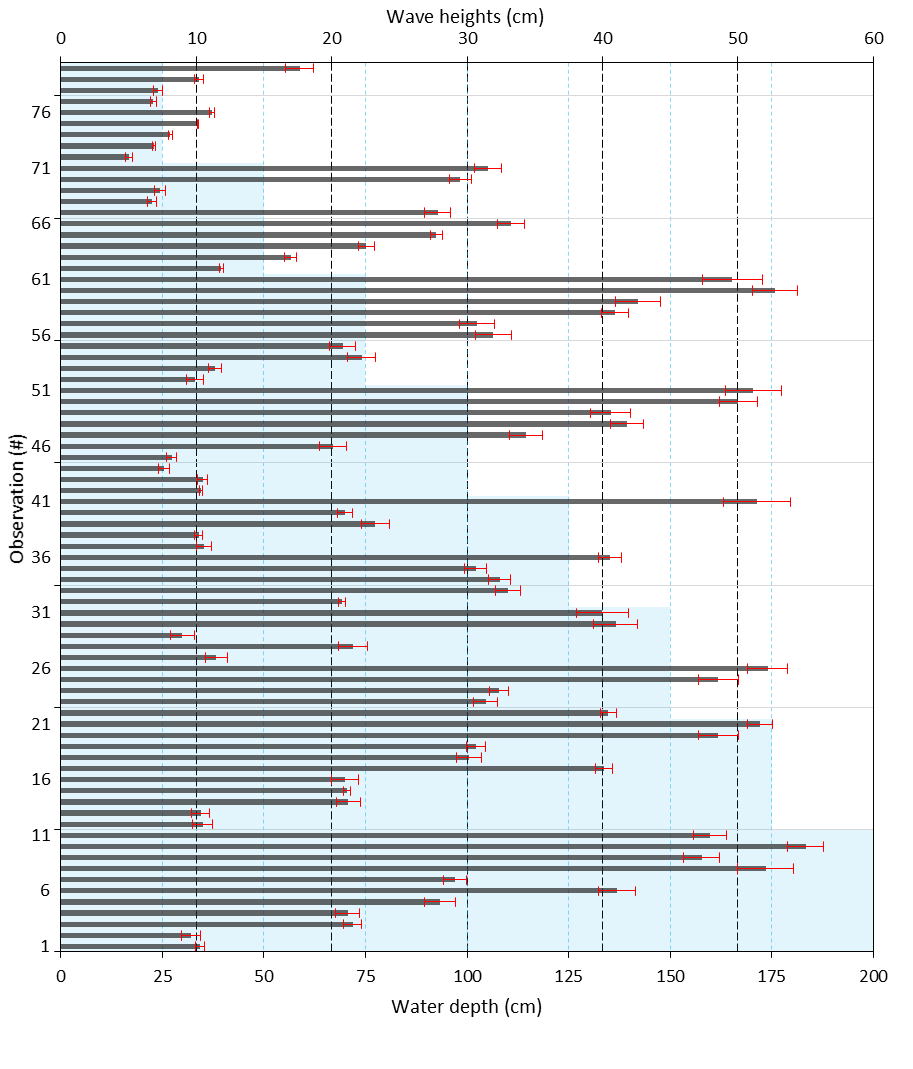


**Figure S.2** Uncertainties in wave height measurements vs. water depth


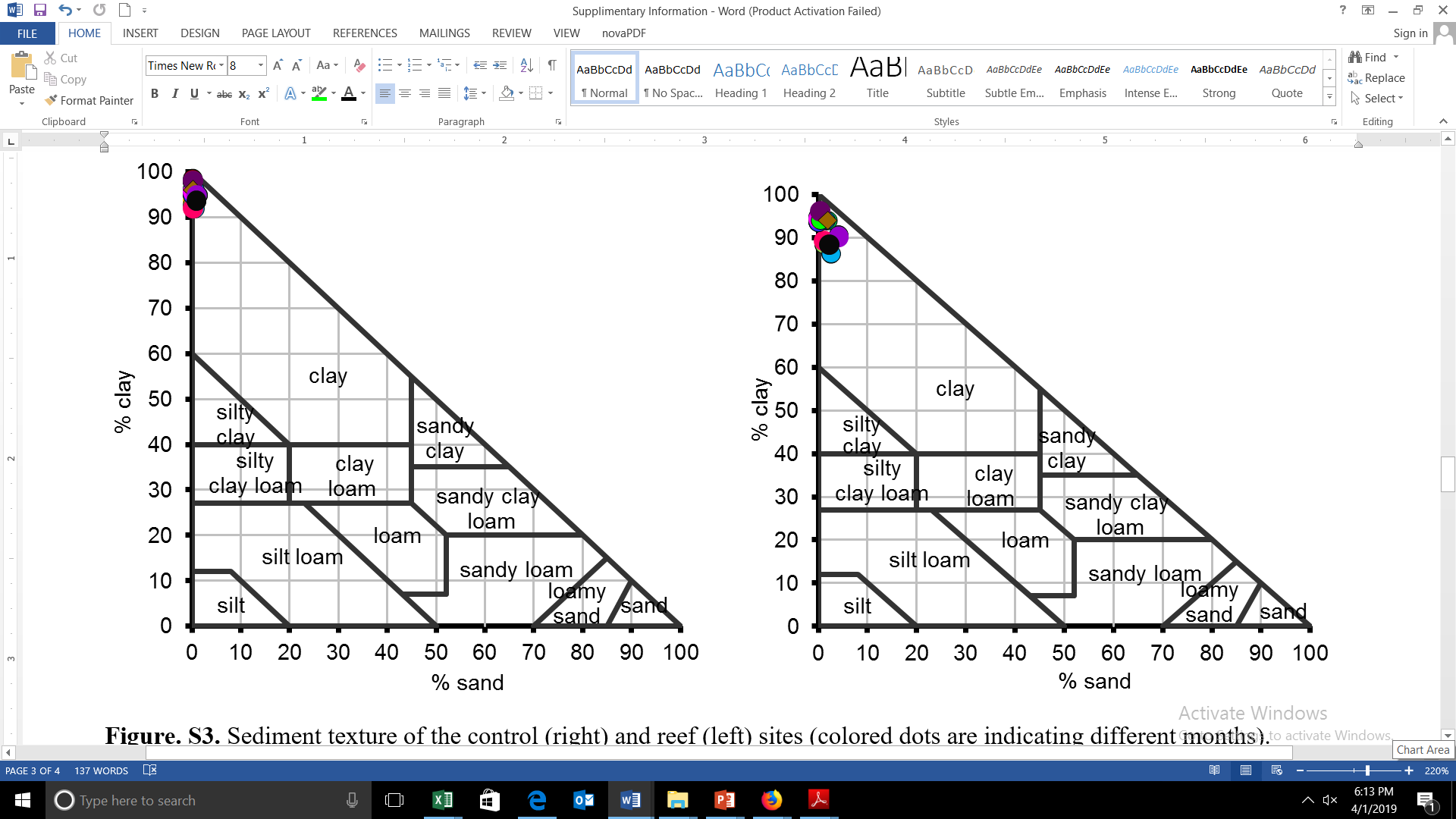


**Figure. S3.** Sediment texture of the control (right) and reef (left) sites (colored dots are indicating different months).


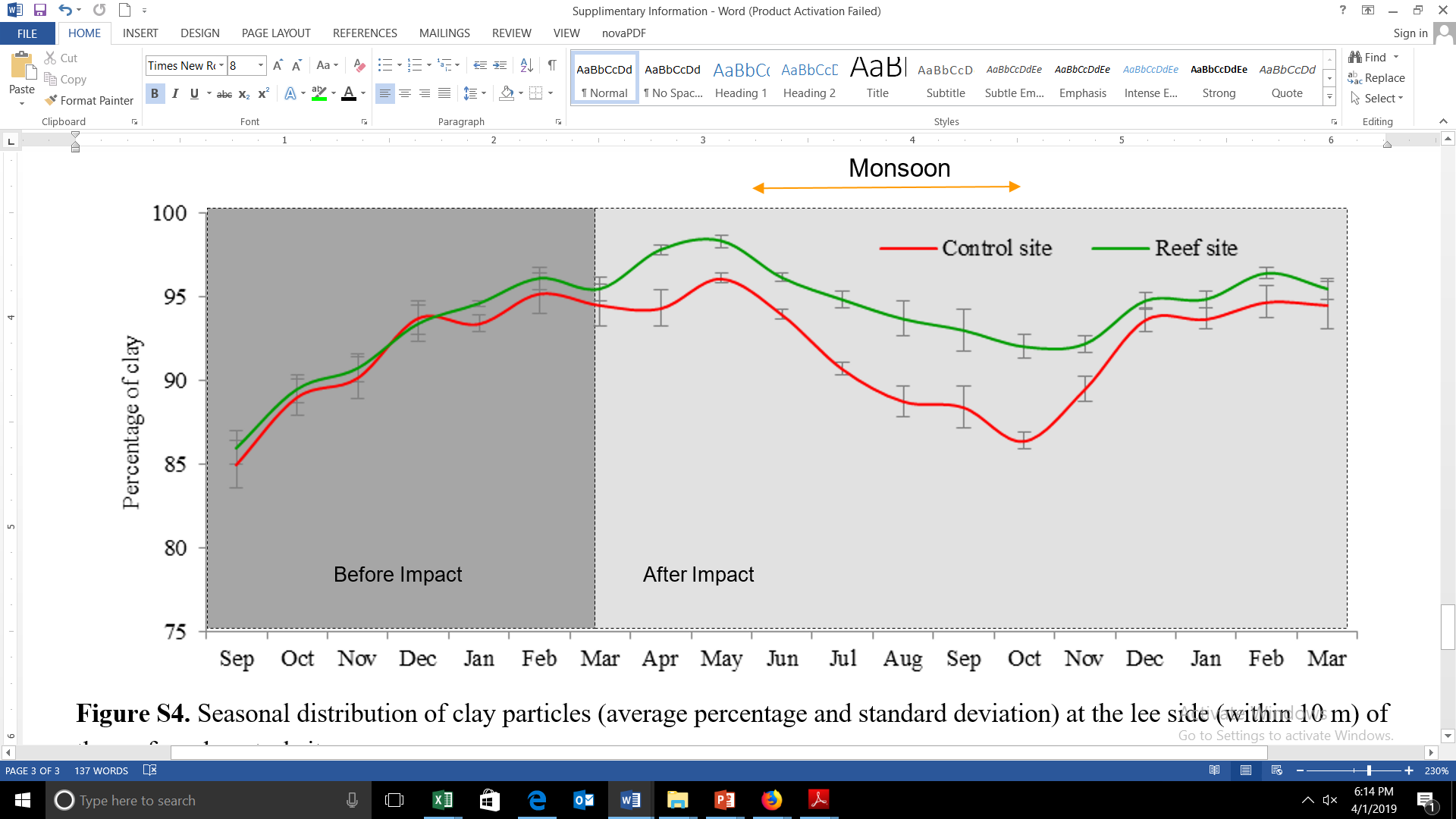


**Figure S4.** Seasonal distribution of clay particles (average percentage and standard deviation) at the lee side (within 10 m) of the reefs and control site.

*
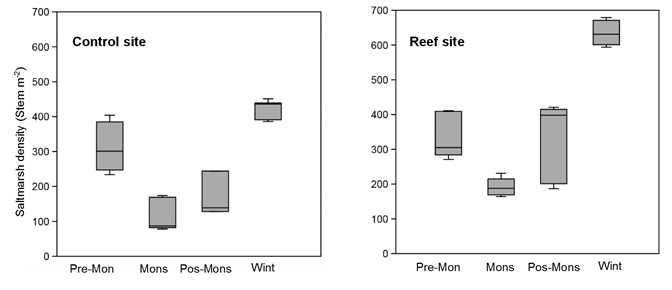
*

**Figure S5.** Seasonal dynamics in saltmarsh density (average and quartiles) in control (left) and reef (right) sites.
